# The impact of radiomics in the management of soft tissue sarcoma

**DOI:** 10.1007/s12672-024-00908-2

**Published:** 2024-03-05

**Authors:** Riccardo De Angelis, Roberto Casale, Nicolas Coquelet, Samia Ikhlef, Ayoub Mokhtari, Paolo Simoni, Maria Antonietta Bali

**Affiliations:** 1 Institut Jules Bordet, Anderlecht, Belgium; 2 Université Libre de Bruxelles, Brussels, Belgium

## Abstract

**Introduction:**

Soft tissue sarcomas (STSs) are rare malignancies. Pre-therapeutic tumour grading and assessment are crucial in making treatment decisions. Radiomics is a high-throughput method for analysing imaging data, providing quantitative information beyond expert assessment. This review highlights the role of radiomic texture analysis in STSs evaluation.

**Materials and methods:**

We conducted a systematic review according to the Systematic Reviews and Meta-Analyses (PRISMA) guidelines. A comprehensive search was conducted in PubMed/MEDLINE and Scopus using the search terms: ‘radiomics [All Fields] AND ("soft tissue sarcoma" [All Fields] OR "soft tissue sarcomas" [All Fields])’. Only original articles, referring to humans, were included.

**Results:**

A preliminary search conducted on PubMed/MEDLINE and Scopus provided 74 and 93 studies respectively. Based on the previously described criteria, 49 papers were selected, with a publication range from July 2015 to June 2023. The main domains of interest were risk stratification, histological grading prediction, technical feasibility/reproductive aspects, treatment response.

**Conclusions:**

With an increasing interest over the last years, the use of radiomics appears to have potential for assessing STSs from initial diagnosis to predicting treatment response. However, additional and extensive research is necessary to validate the effectiveness of radiomics parameters and to integrate them into a comprehensive decision support system.

## Introduction

Soft tissue sarcomas (STSs) are rare malignancies that arise from mesenchymal cells [1]. They account for about 1% of all adult cancers and have a wide range of histological subtypes. Pre-therapeutic tumour grading and assessment are crucial in making treatment decisions, as they provide prognostic information and guide the choice of the proper approach (surgery, chemotherapy, and radiotherapy) [2].

In diagnosing suspected STSs, essential imaging techniques such as ultrasound and Magnetic Resonance Imaging (MRI) are fundamental, with MRI being vital for comprehensive evaluation. Thoracic Computed Tomography (CT), or PET-CT scans are instrumental in identifying metastatic sites, while precise imaging is key for biopsy guidance to accurately localise the lesion [3].

**Fig. 1:**
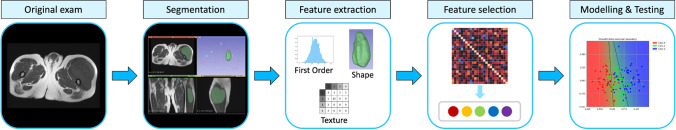
An illustrative radiomics workflow applied to a left thigh myxoid fibrosarcoma

One of the most recenty introduced techniques in radiological science is Radiomics, a high-throughput approach for the analysis of imaging data; this method offers quantitative information that augments expert assessments [4, 5]. It involves the extraction of a large number of features from medical images that reflect the tumour characteristics such as shape, size, intensity, texture, and heterogeneity [6]. Radiomics has been applied to various types of cancers, including STSs, with promising results in terms of diagnosis, prognosis, and prediction [7, 8]. Figure 1 depicts an illustrative radiomics workflow applied to a left thigh myxoid fibrosarcoma, sourced from an open-source anonymized database (10.7937/K9/TCIA.2015.7GO2GSKS) [9, 10].

This review aims to provide an overview of recent publications in the field of STS radiomics. It categorizes these studies into various domains of interest, highlighting the diverse applications and limitations of radiomic analysis in STS.

## Materials and methods

We conducted a systematic review according to the Preferred Reporting Items for Systematic Reviews and Meta-Analyses (PRISMA) guidelines [11]. A comprehensive search was conducted in PubMed/MEDLINE and Scopus using the search terms: ‘radiomics [All Fields] AND ("soft tissue sarcoma" [All Fields] OR "soft tissue sarcomas" [All Fields])’. After removing duplicates, original published articles were included in the analysis.

All single, comparative studies, and primary studies that met the following PICO criteria were selected: P (patients): Patients with STSs; I (interventions): Radiomics; C (comparison): Conventional diagnostic imaging (including CT, MRI,PET/CT); O (outcome): The impact of radiomics on STSs on diagnosis, prognosis, risk stratification, genetic/histological prediction and technical feasibility aspects.

The following exclusion parameters were applied: (1) not original articles (e.g. letters, reviews, editorials, book chapters, congress communications); (2) papers not concerned radiomics topic; (3) researches not referred to humans (e.g. STSs in mice); (4) only articles in English, French, Spanish, Italian or German were included.

Two radiologists (RC, RDE) initially analysed all articles. An independent validation was performed by one other radiologist (MAB), by one resident in radiology (AM), and by one physicist (NC). The complete procedure, along with the results and any discussion regarding probable inconsistencies, was verified by one other independent radiologist (PS), expert in the field of musculo-skeletal oncological radiology.

The quality assessment of the eligible articles was evaluated using the Radiomics Quality Score (RQS) [12] by 2 evaluators (RC) and (SI). Each of the 16 essential criteria specified by the RQS was individually rated, yielding a composite score ranging from -8 to 36 points. These scores were subsequently transformed into RQS percentages, with a score of -8 to 0 points corresponding to 0% and a score of 36 points corresponding to 100% [12].

## Results

**Fig. 2:**
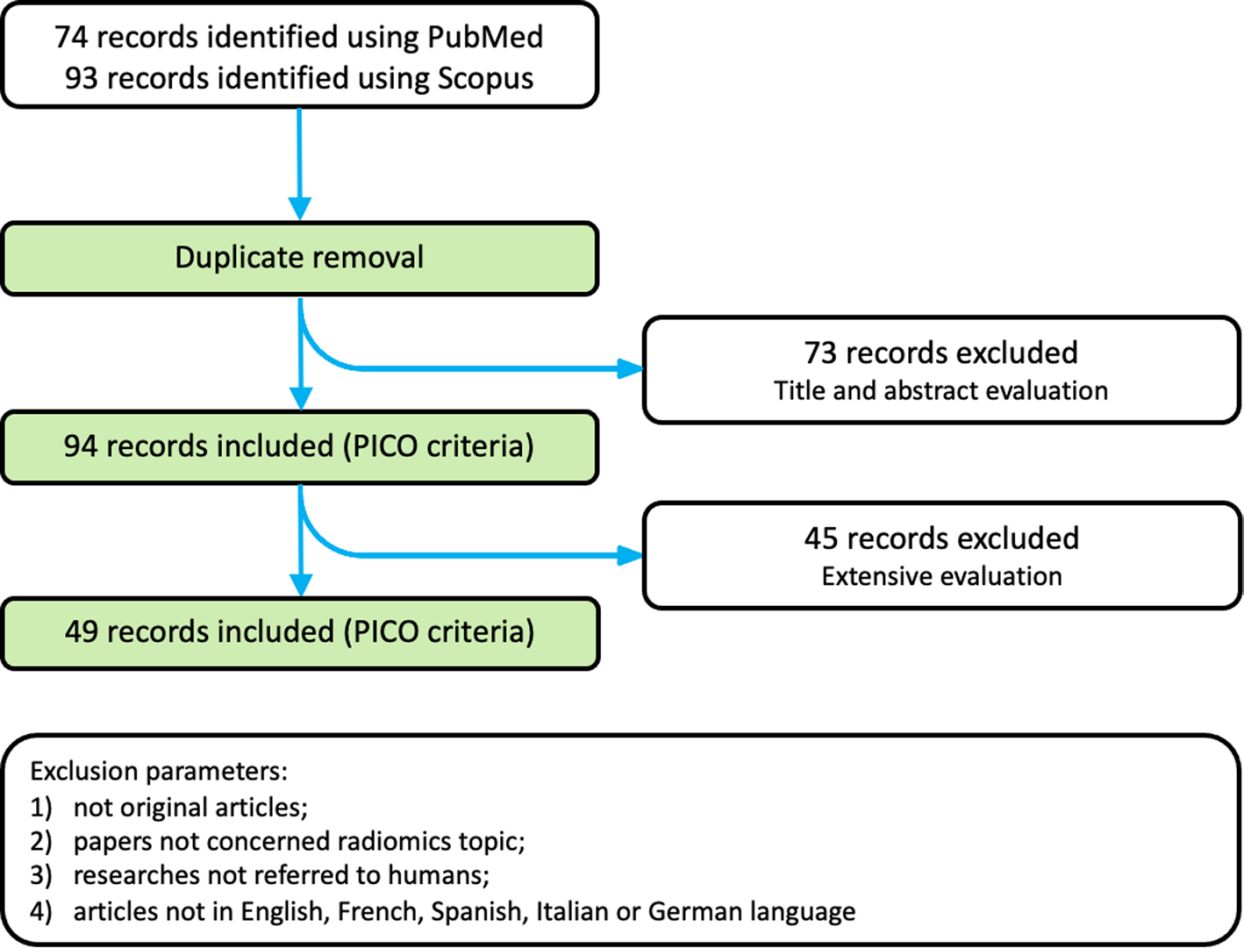
Selection process of literature

A preliminary search conducted on PubMed/MEDLINE and Scopus provided 74 and 93 studies respectively. After removing duplicates and applying the aforementioned PICO criteria, a total of 94 papers were retained through evaluation of their titles and abstracts. Finally, after an extensive selection process (Fig. 2), 49 papers were eligible for analysis. In particular, 36 papers were excluded from consideration due to their non-original article status; 6 papers were omitted as they did not pertain to the subject of radiomics; 4 papers were excluded as they did not involve human subjects; 2 papers were disregarded based on their language of publication.

**Fig. 3:**
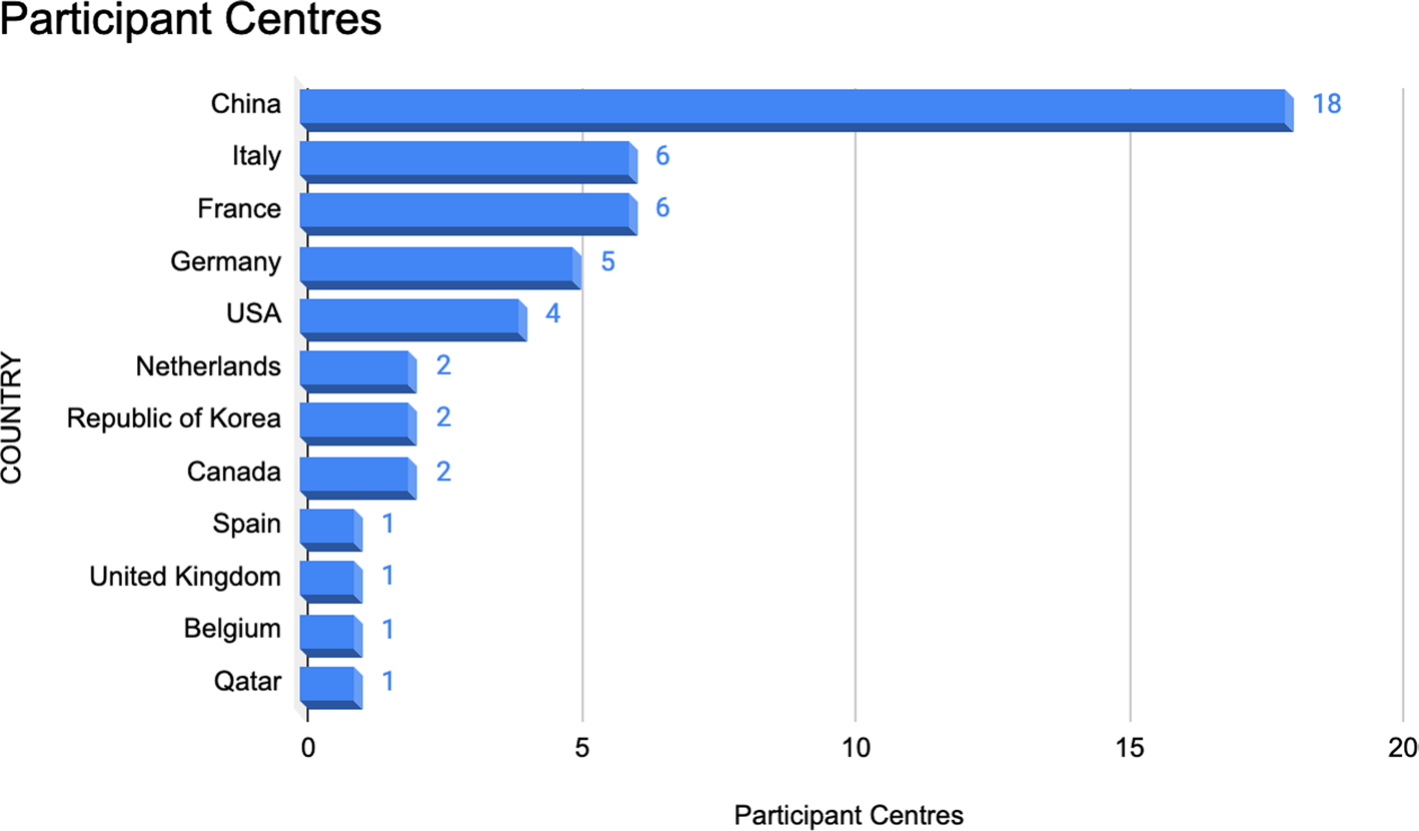
Participant centres of included articles

**Fig. 4:**
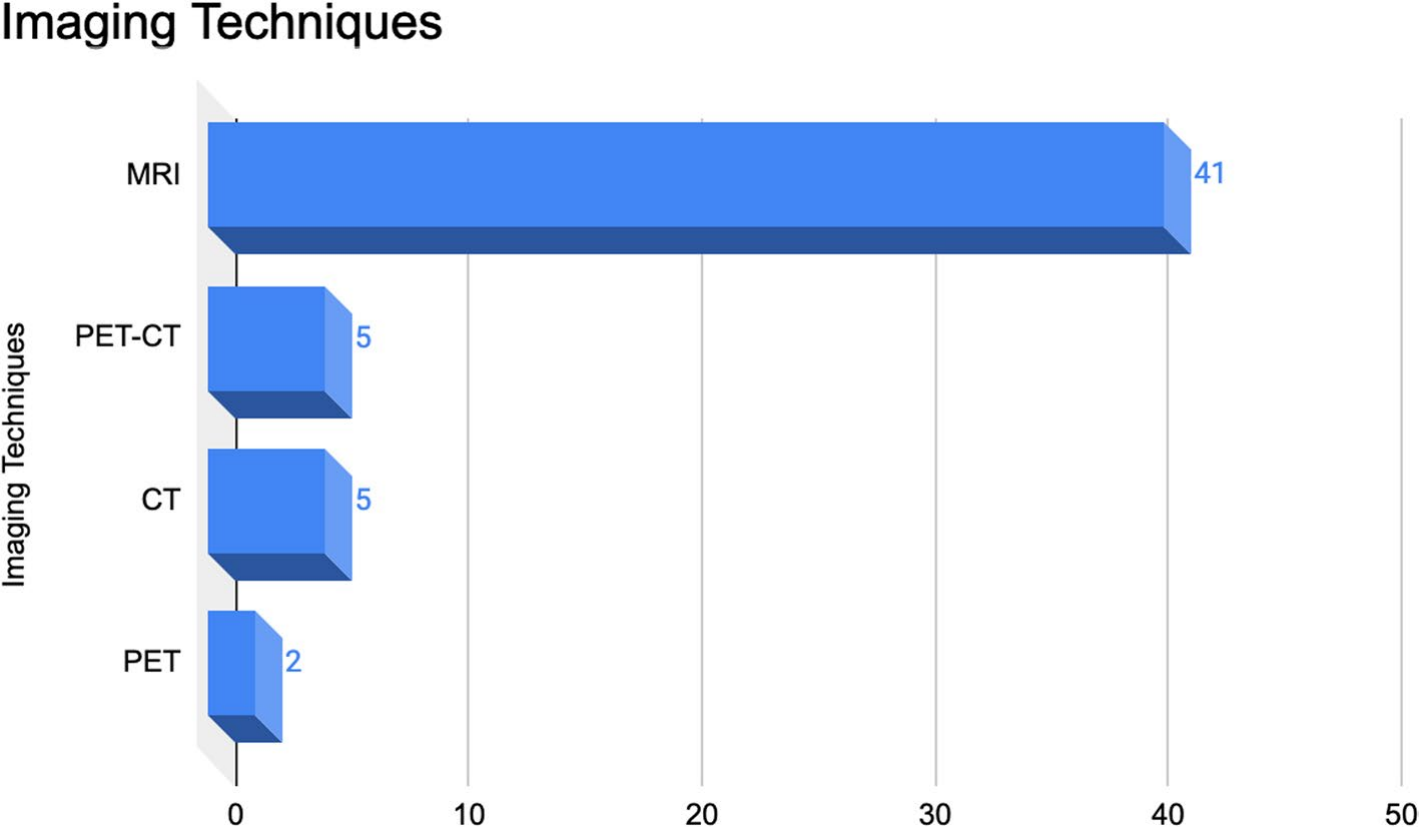
Imaging techniques of included articles

Among the retained papers, 46 studies were retrospective, and 3 studies were prospective, with a publication range from July 2015 to June 2023. The participant centres (Fig. 3) included China (n = 18; 36.7%), Italy (n = 6; 12.2%), France (n = 6; 12.2%), Germany (n = 5; 10.2%), USA (n = 4; 8.2%), Canada (n = 2; 4.1%), Netherlands (n = 2; 4.1%), Republic of Korea (n = 2; 4.1%), Spain (n = 1; 2%), United Kingdom (n = 1; 2%), Belgium (n = 1; 2%), Qatar (n = 1; 2%). MRI (n = 41; 77.4%), PET-CT (n = 5; 9.4%), CT (n = 5; 9.4%) or PET (n = 2; 3.8%) images were used for radiomics analysis (Fig. 4)—some of above-mentioned articles used more than one imaging techniques. The median number of patients involved in the analysis was 63 (range 11–540). The median number of radiomics features was 160 (range 30–2758).

**Fig. 5:**
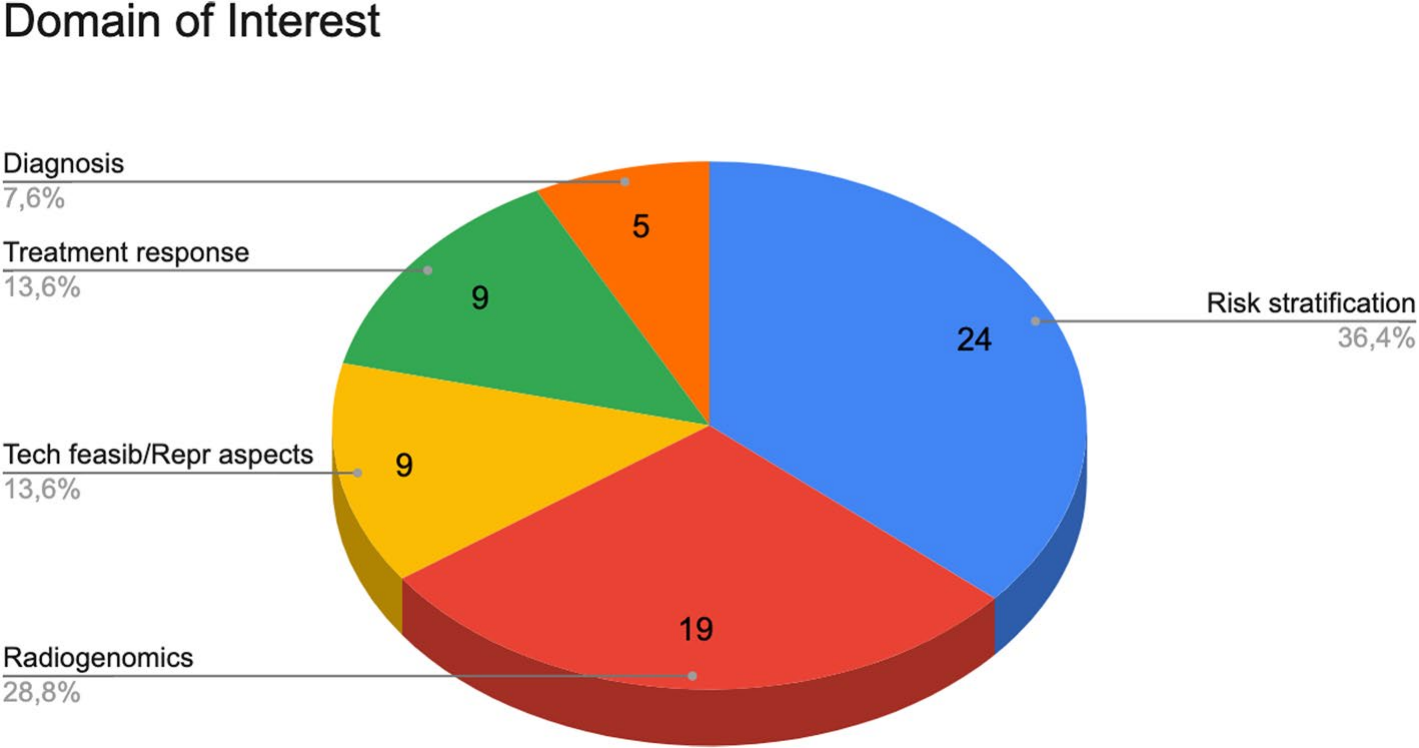
Domains of interest covered by the selected articles

Regarding the domain of interest (Fig. 5): 24 (36.4%) articles were focused on risk stratification, 19 (28.8%) articles on radiogenomics, 9 (13.6%) articles on technical feasibility/reproductive aspects, 9 (13.6%) articles on treatment response and 5 (7.6%) articles on diagnosis—some of aforementioned articles treated more than one domain of interest.

In total, 24 studies analysed the use of radiomics for risk stratification. 7 articles assessed the role of radiomics models for predicting lung metastasis [9, 13–18]; 3 articles analysed radiomics models for prediction of distant metastasis or metastatic relapse-free survival [19–21]. The ability to predict overall survival or free survival was evaluated in 6 studies [22–27]; in particular, according to Spraker et al. [26], texture features related to histogram_skewness, histogram_kurtosis, GLZSM_Small zone/low grey emphasis and GLZSM_Zone, obtained from T1-weighted contrast-enhanced images, were selected in the models for predicting overall survival. Fadli et al. [28] found that increase in heterogeneity (visually evaluated) and logarithmic change in radiomics features clusters, in contrast enhancement MRI T1-weighted images, were independent predictors for metastatic relapse-free and local relapse-free survival. One study developed a radiomics model for predicting disease-free survival in patients with STSs of the extremities and trunk who have undergone neoadjuvant radiotherapy [29]. Lee et al. [30] investigated the effectiveness of a radiomics model using T2-weighted Dixon sequence in differentiating the degree of STSs margin infiltration. Zhao et al. [31] evaluated the ability of various PET/MRI fusion methods to extract features for the prediction of recurrence/metastasis in patients with STSs. Tagliafico et al. [32] analysed MRI radiomics features in surveillance of local recurrence in patients with limb STSs. Liu et al. [33] evaluated the accuracy of two deep learning-radiomic nomogram models, in conjunction with clinical parameters, for predicting local recurrence in patients with STSs who underwent surgical resection. Lastly, one recent study presented a methodology employing MRI radiomic features for the prediction of metastasis and recurrence risk in patients with extremity STSs using formal logic models [34].

19 radiogenomics articles aimed to establish a connection among image phenotype, gene expression, mutations, molecular, or pathological findings. In particular, 11 studies have been conducted to develop and evaluate MRI-based radiomics for the differentiation of grade in STS tumours [20, 25, 32, 35–43]; furthermore, a study among these aforementioned ones aimed to predict the grade and the Ki-67 expression level by utilising intravoxel incoherent motion MRI and diffusion kurtosis imaging parameter maps [43]. Corino et al. [42] discovered that the GLCM features related to dissimilarity and entropy showed higher values in the high-grade. Peeken et al. [44] further explored the potential of quantitative imaging features in CT radiotherapy planning for predicting the grading. In children, using T2-weighted MRI images, Giraudo et al. [45] discovered that lmc1 feature was associated with high-grade tumours and variance feature was associated with rhabdomyosarcomas histotype. One study [27] assessed the predictive value of FDG PET/CT conventional metrics and textural features in determining histopathological data; in particular, the FNCLCC score (representing a histologic surrogate for tumour aggressiveness) demonstrated a correlation with GCLM_dissimilarity, GLCM_contrast and an inverse correlation with GLCM_homogeneity. Crombé et al. [46] investigated the association between distinct patterns of natural evolution of STSs, based on MRI radiomics features, and differential gene expression. Timbergen et al. [47] assessed whether radiomics can differentiate between desmoid-type fibromatosis and STSs and can predict CTNNB1 mutation types in desmoid-type fibromatosis patients. One study [28] examined the associations between temporal alterations observed in MRI, based on qualitative/semi-quantitative features and radiomics features, and the survival outcomes and histopathological characteristics. Foreman et al. [48] developed radiogenomic models with the purpose of predicting the MDM2 gene amplification status and distinguishing between atypical lipomatous tumours and lipomas based on preoperative MRI scans. Nine studies assessed the role of radiomics features for evaluating the toxicity, management and treatment responses of patients with STSs treated with radiotherapy and/or chemotherapy [23, 44, 49–55].

There were 9 studies conducted to assess the technical feasibility and reproducibility of radiomics analysis [9, 16, 17, 31, 35, 55–58]. The influence of ComBatHarmonization on MRI-based radiomics models to differentiate between low-grade and high-grade STS tumours was analysed by Peeken et al. [35]. Thrussell et al. [58] evaluated the repeatability of radiomic features from Diffusion Weighted Imaging (DWI) and Apparent Diffusion Coefficient (ADC) maps in retroperitoneal STSs and compared their repeatability before and after radiotherapy. Vallières et al. [9, 16] assessed the influence of different extraction parameters and acquisition protocols on FDG-PET/MRI models to predict the risk of lung metastasis. Zhao et al. [31] evaluated the ability of various PET/MRI fusion methods to extract features for the prediction of recurrence/metastasis. Sheen et al. [17] analysed the efficacy of four segmentation methods in defining radiomics signatures and prediction models for lung metastases using PET-CT in STSs.

In 5 studies, there were observed associations between imaging features and the ability to diagnose and differentiate STSs from normal tissue or non-malignant lesions. Yue et al. [59] developed a clinical-MRI radiomics nomogram aimed at distinguishing between benign and malignant soft-tissue tumours. Tagliafico et al. [32] investigated the potential use of radiomics in MRI surveillance in patients with limb STSs to differentiate between normal tissue and local recurrence. In another study, Timbergen et al. [47] evaluated MRI radiomics models for distinguishing between desmoid-type fibromatosis and STSs. As previously mentioned, Foreman et al. [48] attempted to develop radiogenomic models with the goal of distinguishing between atypical lipomatous tumours and lipomas by analysing the MDM2 gene amplification status using preoperative MRI scans. Aouadi et al. [60] examined several datasets, in particular the LIPO dataset [61] for distinguishing between well-differentiated liposarcoma and lipoma, and the Desmoid dataset [61] for differentiating desmoid-type fibromatosis from extremity STSs.

Table 1 presents a comprehensive overview of all the aforementioned analysed studies, highlighting the primary objectives, outcomes, and various domains of interest.

**Table 1:** Detailed summary of the analyzed studies, outlining their objectives, conclusions, and diverse domains of interest

DOI	First author (country)	Date	Cluster section	Experimental design	Imaging modality	No. of patients	No. of features	Objectives	Conclusions
10.1088/0031-9155/60/14/5471	Vallières et al (Canada) [9]	Jul 2015	Risk stratification and Technical feasibility/reproductive aspects	Retrospective	PET-CT, MRI	51	50	To develop a texture-based model using FDG-PET and MRI scans to assess the risk of lung metastasis in STSs at an early stage. To evaluate the influence of different extraction parameters on the predictive value of features	The best performing model, from fused scans, achieved an Area Under the receiver operator characteristic Curve (AUC) of 0.984 and a sensitivity of 0.955 in identifying lung metastasis risk. The size of isotropic voxel had the greatest impact on the predictive value
10.1088/1361-6560/aa8a49	Vallières et al (Canada) [16]	Oct 2017	Risk stratification and Technical feasibility/reproductive aspects	Retrospective	PET-CT, MRI	30	55	To investigate the potential to improve a radiomics model for predicting lung metastases development in STS patients; in particular, the study used computer simulations to optimise PET and MRI acquisition protocols with varying parameters	The study found that optimising image acquisition parameters can improve the predictive performance of radiomics models. The model constructed with optimised acquisition parameters showed a significant increase in performance (AUC of 0.89) compared to standard clinical acquisition parameters (AUC of 0.84)
10.1002/jmri.25791	Corino et al (Italy) [42]	March 2018	Radiogenomics	Retrospective	MRI	19	65	To evaluate the ability of MRI radiomics models to classify the grading of STS	Using a few radiomic features, particularly first-order statistics, the model distinguished intermediate- from high-grade STSs (AUC of 0.87)
10.1007/s10278-018-0092-9	Bologna et al (Italy) [57]	Dec 2018	Technical feasibility/reproductive aspects	Retrospective	MRI	36	69	To evaluate the stability and discrimination capability of radiomic features, applying translations of regions of interests in ADC maps extracted from DWI. STS patients and oropharyngeal cancers were analysed in this study	Using intraclass correlation coefficient, 54 radiomics features for oropharyngeal cancers and 59 for STSs were found discriminative and stable; in particular, the stability depended on the region of the body under evaluation
10.1016/j.adro.2019.02.003	Spraker et al (USA) [26]	Febr 2019	Risk stratification	Retrospective	MRI	226	30	To investigate whether using radiomic features extracted from MRI could predict overall survival in patients with stage II-III STSs	The best-performing model (built on 5 radiomics features, age and grade) obtained a C-index of 0.78 in the validation cohort
10.1016/j.radonc.2019.01.004	Peeken et al (Germany) [44]	Jun 2019	Treatment response and Radiogenomics	Retrospective	CT	221	1358	To determine if quantitative imaging features of radiotherapy planning CT-scans can be used for predicting the grading and for assessing the pre-therapeutic risk (overall survival, distant progression free survival, local progression free survival) in STS patients	The radiomic grading model distinguished grade 3 from non-grade 3 STSs with a maximum AUC of 0.65. The combined clinical-radiomic model obtained a C-index of 0.75 and 0.76 for overall survival, C-index of 0.60 and 0.68 for distant progression free survival, and C-index of 0.62—0.71 for local progression free survival (respectively in two validation cohorts)
10.1002/jmri.26589	Crombé et al (France) [52]	Aug 2019	Treatment response	Retrospective	MRI	65	33	To investigate if a delta-radiomics approach can improve early response prediction in patients with high-grade STSs undergoing neoadjuvant chemotherapy	The study found that a delta-radiomics approach, based on changes in texture and shape features, provided higher diagnostic performance for early response prediction compared to RECIST criteria and semantic radiological variables (except for edema decrease). The best model, built on three features, provided an AUC of 0.746, but a specificity of 28% on the test cohort
10.1016/j.acra.2018.09.025	Zhang et al (China) [36]	Sep 2019	Radiogenomics	Retrospective	MRI	35	1049	To develop a radiomics model that can predict the histopathological grades of STSs before surgery using MRI	This study found that the radiomics model based on five quantitative imaging features extracted from Fat-Suppressed T2-weighted (T2FS) showed accuracy of 0.88 and AUC of 0.92 in predicting the histopathological grades of STSs noninvasively
10.2478/raon-2019-0041	Tagliafico et al (Italy) [32]	Sep 2019	Radiogenomics and Risk stratification and Diagnosis	Retrospective	MRI	11	104	To investigate the use of radiomics analysis in MRI surveillance in patients with limb STSs, and to identify radiomics features that can differentiate between normal tissue and local recurrence	Four different radiomics features showed a significant correlation with the size of the tumour; four other radiomics features were found to be correlated with the grading of the tumour. For differentiation of normal tissue versus local recurrence, the Receiver Operating Characteristic (ROC) analysis revealed an AUC ranging from 0.71 for T1-weighted to 0.96 for post-contrast T1-weighted
10.1016/j.ebiom.2019.08.059	Peeken et al (Germany) [35]	Oct 2019	Radiogenomics and Technical feasibility/reproductive aspects	Retrospective	MRI	225	1394	To develop MRI-based radiomics grading models to differentiate between low-grade and high-grade STS tumours. To analyse the influence of ComBatHarmonization on validation performance	The radiomic model based on T2FS obtained predictive performances with an AUC of 0.78 on the independent validation set. Combining the radiomics model with clinical staging improved prognostic performance and net benefit
10.1002/jmri.26753	Crombé et al (France) [55]	Dec 2019	Treatment response and Technical feasibility/reproductive aspects	Prospective	MRI	25	32	To analyse how temporal factors affect texture features, in dynamic contrast-enhanced MRI parametric maps, for measuring intratumoral heterogeneity in STSs, and to evaluate the ability of models in predicting the response to chemotherapy	Temporal resolution influenced several features extracted from area under time-intensity curve and Ktrans maps; scan duration influenced various features extracted from Ktrans maps. The models based on baseline texture features showed predictive performance for response to chemotherapy, with AUCs ranging from 0.77 to 0.90
10.1002/jmri.26901	Wang et al (China) [40]	March 2020	Radiogenomics	Retrospective	MRI	113	556	To evaluate the effectiveness of radiomics features in differentiating histological grades of STSs	The study demonstrated that a machine-learning model based on recursive feature elimination and Random Forest (RF) classification algorithms, combined with synthetic minority oversampling, achieved the best performance in predicting the grade of STSs, with an AUC of 0.9615 in the validation dataset
10.1148/radiol.2020191145	Zwanenburg et al (Germany) [56]	May 2020	Technical feasibility/reproductive aspects	Retrospective	PET-CT, MRI	51	174	To standardise a set of radiomic features using a digital phantom, CT images of a patient with lung cancer and a data set of multimodality images from 51 patients with STS	A total of 169 radiomics features were successfully standardised, allowing for verification and calibration of different radiomics software
10.1155/2020/8153295	Deng et al (China) [18]	May 2020	Risk stratification	Retrospective	PET	51	67	To assess a feature fusion method derived from clinical data and PET images for predicting lung metastasis of STSs	The proposed feature fusion method, using 3 features from PET and 7 clinical features, obtained better prediction ability for lung metastasis compared to single-mode methods. The model achieved an average accuracy of 0.92
10.1002/jmri.27040	Crombé et al (France) [21]	Jul 2020	Risk stratification	Retrospective	MRI	50	92	To determine the most effective method for predicting metastatic relapse-free survival from baseline MRI in nonmetastatic high-grade STSs, comparing a classical semantic radiological model, a conventional radiomics model based on T2, and models depending on the postprocessing of dynamic contrast enhanced MRI	The most accurate models included all relative changes in radiomics features and integrated relative changes in radiomics features with a C-index of 0.83. The classical semantic radiological model obtained the highest integrative AUC (0.87)
10.1088/1361-6560/ab9e58	Gao et al (USA) [54]	Aug 2020	Treatment response	Retrospective	MRI	30	106	To examine the potential of radiomics features obtained from longitudinal DWI MRI to predict treatment response in patients with localised STSs who received hypofractionated preoperative radiotherapy	Using features from all time points and corresponding delta radiomics, the Support Vector Machine (SVM) model reached an AUC of 0.91 for the prediction of treatment response
10.2214/AJR.19.22147	Xu et al (China) [37]	Oct 2020	Radiogenomics	Retrospective	MRI	105	792	To evaluate the effectiveness of radiomics features in distinguishing histopathologic grades of STSs	The combination of the RF classification algorithm with the Least Absolute Shrinkage and Selection Operator (LASSO) feature selection method reached an accuracy of 0.9143 for the prediction of STS grade in the validation set
10.1016/j.ejrad.2020.109266	Timbergen et al Netherlands[47]	Oct 2020	Diagnosis and Radiogenomics	Retrospective	MRI	203	411	To assess whether radiomics can differentiate between desmoid-type fibromatosis and STSs and predict CTNNB1 mutation types in desmoid-type fibromatosis patients	The T1-weighted radiomics model, in combination with age and sex, reached an AUC of 0.88 in distinguishing desmoid-type fibromatosis from STS and an AUC of 0.74 in predicting CTNNB1 mutation status
10.1016/j.crad.2020.08.038	Tian et al (China) [19]	Febr 2021	Risk stratification	Retrospective	MRI	77	160	To develop and validate a radiomics-based machine learning model that can predict the likelihood of distant metastasis from STSs before surgery	The study showed that the combination of LASSO and SVM algorithms with SMOTE improved the performance of the machine-learning model, which had an accuracy of 0.91 and an AUC of 0.902 in the validation dataset
10.3390/cancers13081929	Peeken et al (Germany) [24]	April 2021	Risk stratification	Retrospective	MRI	179	105	To investigate the value of MRI-based radiomics and expert-derived semantic imaging features in predicting overall survival in patients with STSs of the extremities	Radiomic models based on patients’ age, AJCC staging and Radiomics-T2-weighted obtained the best performance in the test set (C-index: 0.73)
10.1002/jmri.27532	Yan et al (China) [39]	Jun 2021	Radiogenomics	Retrospective	MRI	180	1793	To generate and validate a noninvasive MRI based radiomics nomogram for predicting the grade of STSs	The radiomics nomogram, which incorporated significant risk factors and the radiomics signature, demonstrated good performance in predicting the grade of STSs, with an AUC of 0.916 in the training set and an AUC of 0.879 in the external validation set
10.5603/RPOR.a2021.0092	González-Viguera et al (Spain) [23]	Sep 2021	Risk stratification and Treatment response	Retrospective	CT	25	44	To assess the management, toxicity, and treatment responses of patients with STSs treated with neoadjuvant radiotherapy, and to investigate the potential use of CT radiomics features	The study found an association between CT radiomics features and various outcomes. Specifically, GLCM_correlation was associated with local control; while HUmin, HUpeak, volume, GLCM_correlation, and GLZLM_GLNU were associated with systemic control. Additionally, GLZLM_SZE was associated with overall survival
10.3389/fonc.2021.710649	Chen (China) [29]	Sep 2021	Risk stratification	Retrospective	MRI	62	851	To develop a radiomics model for predicting disease-free survival in patients with STSs of the extremities and trunk who have undergone neoadjuvant radiotherapy	The combined clinical-radiomics model obtained the best predictive ability for disease-free survival (C-index: 0.781; AUC: 0.791)
10.1016/j.radonc.2021.08.023	Peeken et al (Netherlands) [50]	Novemb 2021	Treatment response	Retrospective	MRI	156	105	To investigate whether temporal changes in radiomic features following neoadjuvant treatment ("delta-radiomics") can predict the pathological complete response in high-grade STSs more accurately than pretreatment radiomic models	The combined delta-radiomics achieved the best value (AUC of 0.75) and outperformed conventional predictors such as tumour volume and RECIST in predicting the pathological complete response
10.1186/s40644-021-00438-y	Zhao et al (China) [31]	Jan 2022	Risk stratification and Technical feasibility/reproductive aspects	Retrospective	MRI	51	136	To evaluate the ability of various PET/MRI fusion methods (image-level fusion, feature-level fusion, and matrix-level fusion) to extract features for the prediction of recurrence/ metastasis in patients with STSs	Image-level fusion method, using discrete wavelet transformation, showed the best classification performance among the fusion methods tested (optimal AUCs for the T1/PET image-level fusion was 0.9524 and for the T2/PET image-level fusion 0.9048)
10.1007/s40042-021-00360-3	Sheen et al (Republic of Korea) [17]	Jan 2022	Risk stratification and Technical feasibility/reproductive aspects	Retrospective	PET	48	44	To investigate the efficacy of four segmentation methods in defining radiomics signatures and prediction models for lung metastases of STSs using PET-TC	The GLRLM_RLNU, obtained from all segmentation methods, was identified as a meaningful radiomics feature associated with tumour heterogeneity and aggressiveness. The best model, based on gradient-based edge detection segmentation methods, achieved the best AUC (0.88)
10.1007/s13304-021-01074-8	Yang et al (China) [22]	Febr 2022	Risk stratification	Retrospective	CT	353	86	To determine the association between radiomic features and overall survival in patients with extremity and trunk wall STSs using Random Survival Forest analysis, and to compare the performance of this model with Cox proportional hazard model	The Random Survival Forest model, incorporating three important radiomic features and clinical characteristics, demonstrated good predictive performance in overall survival prediction. The model showed little advantage over the Cox proportional hazard model. The C-index in both integrative models fluctuated above 0.80 in the training and testing datasets
10.1007/s00330-021-08221-0	Liu et al (China) [33]	Febr 2022	Risk stratification	Retrospective	MRI	282	1452	To assess the accuracy of two deep learning-radiomic nomogram models, associated with clinical data, in predicting tumour recurrence in patients with STSs who underwent surgical resection	The two deep learning radiomic nomogram models showed a C-index of 0.721 or higher and a median AUC of 0.746 or higher
10.3390/tomography8010028	Tomaszewski et al (USA) [53]	Febr 2022	Treatment response	Retrospective	CT	296	164	To investigate whether computational analysis, of pretreatment imaging data, could identify patients who had a significantly longer overall survival if treated with doxorubicin in combination with evofosfamide vs. only doxorubicin	The study showed that a radiomic criterion, incorporating a single radiomics feature, histological classification, and smoking history, could be used to identify exclusion criteria for patients who were expected to obtain the greatest benefit from treatment with doxorubicin alone
10.3389/fonc.2022.897676	Liang et al (China) [13]	Jun 2022	Risk stratification	Retrospective	MRI	242	1379	To develop and test the performance of a deep learning radiomics nomogram for predicting the lung metastasis status in patients with STSs using radiomics features and clinical predictors	The best model, which combined independent clinical predictors with the best-performing radiomics prediction signature, demonstrated superior predictive performance for lung metastasis status (AUC of 0.833 on the external validation set) compared to the clinical and radiomics models
10.2478/raon-2022-0013	Giraudo et al (Italy) [45]	Jun 2022	Radiogenomics	Retrospective	MRI	11	33	To evaluate the potential diagnostic value of radiomic features extracted from axial T2-weighted images in children with STSs examined by PET/MRI for staging	Specific radiomic features were identified as potential biomarkers; in particular, lmc1 feature obtained accuracy of 0.70 for classifying high grade tumours and variance feature showed accuracy of 0.83 for detecting rhabdomyosarcomas histotype
10.1002/mp.15603	Escobar et al (France) [15]	Jun 2022	Risk stratification	Retrospective	PET-CT, MRI	51	336	To build radiomics models, using MRI and PET-TC, for predicting the risk of lung metastasis and for generating quantitative maps associated with biological patterns	The models were able to identify biological image patterns related to necrosis development and glucose metabolism, which were associated with the risk of lung metastasis
10.3389/fonc.2022.899180	Thrussell et al (United Kingdom) [58]	Jul 2022	Technical feasibility/reproductive aspects	Retrospective	MRI	30	107	To evaluate the repeatability of radiomic features from DWI and ADC maps in retroperitoneal STSs, and to compare their repeatability before and after radiotherapy	The study found that the ADC-based radiomic analysis was more reliable than the features derived from DWI images, and some of these features were sensitive to post-treatment changes
10.1002/jmri.28021	Fadli et al (France) [28]	Jul 2022	Radiogenomics and Risk stratification	Retrospective	MRI	68	85	To examine the associations between temporal alterations observed in MRI, based on qualitative/semi-quantitative features and radiomics features, and the survival outcomes and histopathological characteristics	Alterations in the MRI of STSs, prior to initiation of any therapeutic intervention, were observed. These changes were associated with histopathological findings, and could contribute to patient prognostication
10.1186/s12880-022-00859-6	Miao et al (China) [49]	Jul 2022	Treatment response	Retrospective	CT	51	851	To determine whether radiomics features from contrast-enhanced CT can be used to predict the effectiveness of epirubicin combined with ifosfamide treatment in patients with pulmonary metastases from STSs	The model, developed using CT images before treatment, can be a useful tool for predicting lesion progression and the efficacy of chemotherapy (AUC of 0.856 in the testing group)
10.1186/s12880-022-00876-5	Liu et al (China) [41]	Aug 2022	Radiogenomics	Retrospective	MRI	504	2758	To propose an effective solution for predicting high-grade versus low-grade STSs, using an optimal imbalance machine learning model	The proposed machine learning method (recursive feature elimination technique + SMOTETomek + extremely randomised trees) achieved an AUC of 0.9438, using the dataset splitting method called SRS
10.3389/fonc.2022.879553	Giraudo et al (Italy) [20]	Oct 2022	Radiogenomics and Risk stratification	Retrospective	MRI	36	33	To assess the prognostic role of radiomic variables extracted from ADC maps collected at diagnosis in patients with STSs in terms of overall survival, metastatic spread, and to evaluate the relationship between radiomics features and the tumour grade	The radiomic feature Imc1 was found to be a predictor of metastatic spread in patients with STSs, with an accuracy of 76.7%. The feature also showed a moderate correlation with the tumour grade, while none of the examined variables were predictors of the overall outcome
10.1002/jmri.28518	Yang et al (China) [38]	Novemb 2022	Radiogenomics	Retrospective	MRI	149	1037	To develop and evaluate an MRI-based radiomics nomogram for assessing the Ki-67 status of STSs	The nomogram demonstrated good performance in accurately identifying the Ki-67 status of STSs (C-index of 0.852 in the validation cohort)
10.1002/mp.16136	Hu et al (China) [14]	Novemb 2022	Risk stratification	Retrospective	MRI	154	1967	To investigate the development of MRI-based radiomics models for identifying lung metastasis in STS patients	The study developed a clinical-radiomics nomogram that integrated radiomics features and margin to predict lung metastasis, achieving the best prediction performance with an AUC of 0.800 in the external validation set
10.1007/s11307-022-01751-z	Yue ey al (China) [59]	Dec 2022	Diagnosis	Retrospective	MRI	148	1967	To compare the diagnostic values of a clinical-radiomics nomogram for distinguishing between benign and malignant soft-tissue tumours	The clinical-radiomics nomogram, incorporating radiomic features and clinical factors, demonstrated high diagnostic accuracy for distinguishing between benign and malignant soft-tissue tumours (AUCs of 0.913 in the validation cohort)
10.1002/jmri.28160	Yang (China) [25]	Dec 2022	Radiogenomics and Risk stratification	Retrospective	MRI	540	851 radiomics features + 4096 CNN features	To assess the performance of MRI-based computer-aided diagnostic models in identifying low-grade and high-grade STSs and predicting overall survival	The MRI-based computer-aided diagnostic nomogram (generated by clinical variables, tumour location, size, radiomics and deep learning features) demonstrated an AUC of 0.855 in identifying low-grade and high-grade STSs in the validation cohort. The prognostic model obtained good performance in predicting long-term survival with a 3-year C-index of 0.642 and 5-year C-index of 0.676 in the validation cohort
10.3390/jcm12010279	Annovazzi et al (Italy) [27]	Dec 2022	Radiogenomics and Risk stratification	Retrospective	PET-CT	51	45	To assess the predictive value of FDG PET/CT conventional metrics and textural features in determining histopathological data, free survival and overall survival in patients with undifferentiated STSs of the limbs and trunk	The FNCLCC score (representing a histologic surrogate for tumour aggressiveness) demonstrated a correlation with GCLM_dissimilarity, GLCM_contrast and an inverse correlation with GLCM_homogeneity. In multivariate analysis, GLCM_correlation and perioperative treatment were the only independent factors, affording the categorization of the population into three distinct prognostic categories
10.1007/s11307-023-01803-y	Fields et al (USA) [51]	Jan 2023	Treatment response	Retrospective	MRI	44	1708	To evaluate the performance of machine learning models, based on MRI and delta-radiomics features, in predicting neoadjuvant chemotherapy response in patients with STSs	Although the machine learning models were not able to predict neoadjuvant chemotherapy response (AUC of 0.40–0.44), a subset of Laws Texture Energy derived metrics showed statistical significance in univariate analyses
10.1007/s00330-022-09104-8	Crombé et al (France) [46]	Febr 2023	Radiogenomics and Risk stratification	Retrospective	MRI	63	108	To investigate the association between distinct patterns of natural evolution of STSs, based on MRI radiomics features, and differential gene expression	The study identified three distinct delta-radiomics patient groups, which were associated with different transcriptomic features. In particular, group B showed upregulation of Hedgehog and Hippo signalling pathways and downregulation of natural killer cell-mediated cytotoxicity; while group A demonstrated morphological stability and no local relapse
10.1088/2057-1976/acc33a	Aouadi et al (Qatar) [60]	2023 Mar	Diagnosis	Retrospective	MRI	116 (LIPO dataset) + 203 (Desmoid dataset)	2016	Seven public datasets were analysed to determine the grading classification using radiomic analysis or deep convolutional neural networks. Two datasets were specifically analysed for STSs, the first (LIPO) to classify well-differentiated liposarcoma or lipoma, and the second (Desmoid) to classify desmoid-type fibromatosis or extremity STSs	The best radiomics approach achieved an AUC of 0.86 for the LIPO dataset and 0.844 for Desmoid dataset. The best deep convolutional neural networks approach achieved an AUC of 0.982 for the LIPO dataset and 0.961 for Desmoid dataset
10.1002/jmri.28331	Lee et al (Republic of Korea) [30]	March 2023	Risk stratification	Retrospective	MRI	72	1132	To investigate the effectiveness of a radiomics model using T2-weighted Dixon sequence in differentiating the degree of STSs margin infiltration	The radiomics model constructed with radiomic volume and shape and other T2 features showed the highest AUC (0.821) both for the models generated by LASSO + RF and LASSO + SMOTE + RF algorithms
10.3390/cancers15072150	Foreman et al (Germany) [48]	2023 Apr	Diagnosis and Radiogenomics	Retrospective	MRI	307	312	To build and validate radiogenomic models aimed at predicting the MDM2 gene amplification status and differentiating between atypical lipomatous tumours and lipomas using preoperative MRI scans	The LASSO classifier, utilising radiomic features extracted from all imaging sequences, exhibited excellent performance, achieving an AUC of 0.88 in the testing dataset
10.1093/jamiaopen/ooad025	Casale et al (Belgium) [34]	2023 Apr	Risk stratification	Retrospective	MRI	47	102	To propose a methodology that utilised formal logic models to predict the risk of metastases and recurrence in patients with extremity STSs by analysing MRI radiomic features	The sensitivity and specificity of the methodology were found to be 0.81 and 0.67, respectively
10.1177/02841851231179933	Zhu et al (China) [43]	2023 Jun	Radiogenomics	Retrospective	MRI	42	1409	Radiomics models were developed to predict the histopathological grade and Ki-67 expression level of STSs using intravoxel incoherent motion MRI and diffusion kurtosis imaging MRI parameter maps	In the validation set, the best model (D-SVM) for histopathological grade achieved an AUC of 0.88, and the best model (MK-SVM) for Ki-67 expression level achieved an AUC of 0.83

The 49 articles showed a median RQS of 12 and the values varied from 2 to 23 (Table 2). Additionally, the median RQS expressed as a percentage was 33%, with the minimum recorded value being 6%, while the highest was 64%.

**Table 2:** RQS for the 49 selected articles

**DOI**	First author and year	Image protocol quality	Multiple segmentations	Phantom study	Test–retest (imaging at multiple time points)	Feature reduction or adjustment multiple testing	Multivariate analysis with non-radiomics features	Biological correlates	Cut-off analyses	Discrimination statistics	Calibration statistics	Prospective study	Validation	Comparison to “gold standard”	Potential clinical utility	Cost-effectiveness analysis	Open science and data	**RQS SCORE**	**RQS PERCENTAGE**
10.1088/0031-9155/60/14/5471	Vallières et al. 2015 [9]	2	/	/	/	3	/	1	1	2	/	/	-5	/	2	/	3	9	25
10.1088/1361-6560/aa8a49	Vallières et al. 2017 [16]	2	1	/	/	3	/	1	1	2	/	/	-5	/	2	/	3	10	28
10.1002/jmri.25791	Corino et al. 2018 [42]	2	/	/	/	3	/	1	/	2	/	/	-5	/	2	/	/	5	14
10.1007/s10278-018-0092-9	Bologna et al. 2018 [57]	2	1	/	/	3	/	1	/	1	/	/	-5	/	2	/	/	5	14
10.1016/j.adro.2019.02.003	Sparker et al. 2019 [26]	2	/	/	/	3	1	/	1	2	/	/	3	/	2	/	/	14	39
10.1016/j.radonc.2019.01.004	Peeken et al. 2019 [44]	2	1	/	/	3	1	/	1	2	2	/	4	2	2	/	1	21	58
10.1002/jmri.26589	Crombé et al. 2019 [52]	1	/	/	/	3	/	1	1	2	/	/	2	2	2	/	/	14	39
10.1016/j.acra.2018.09.025	Zhang et al. 2019 [36]	2	/	/	/	3	/	/	/	2	/	/	-5	/	2	/	/	4	11
10.2478/raon-2019-0041	Tagliafico et al. 2019 [32]	2	1	/	/	3	/	1	/	2	/	7	-5	/	2	/	/	13	36
10.1016/j.ebiom.2019.08.059	Peeken et al. 2019 [35]	2	1	/	/	3	/	/	1	2	1	/	3	2	2	/	1	18	50
10.1002/jmri.26753	Crombé et al. 2019 [55]	2	/	/	1	3	/	1	/	1	/	7	-5	/	2	/	/	12	33
10.1002/jmri.26901	Wang et al. 2020 [40]	2	/	/	/	3	/	/	/	2	/	/	2	/	2	/	/	11	31
10.1148/radiol.2020191145	Zwanenburg et al. 2020 [56]	2	1	1	/	3	/	/	/	1	2	/	4	/	2	/	2	18	50
10.1155/2020/8153295	Deng et al. 2020 [18]	2	/	/	/	3	1	/	/	2	/	/	-5	/	2	/	2	7	19
10.1002/jmri.27040	Crombé et al. 2020 [21]	2	/	/	/	3	1	1	1	2	/	/	-5	/	2	/	/	7	19
10.1088/1361-6560/ab9e58	Gao et al. 2020 [54]	2	/	/	/	3	/	1	/	2	/	7	-5	/	2	/	/	12	33
10.2214/AJR.19.22147	Xu et al. 2020 [37]	2	/	/	/	3	/	/	/	2	/	/	2	/	2	/	/	11	31
10.1016/j.ejrad.2020.109266	Timbergen et al. 2020 [47]	1	1	/	/	3	1	/	/	2	/	/	-5	2	2	/	1	8	22
10.1016/j.crad.2020.08.038	Tian et al. 2021 [19]	2	/	/	/	3	/	/	/	2	/	/	2	/	2	/	/	11	31
10.3390/cancers13081929	Peeken et al. 2021 [24]	2	1	/	/	3	1	1	1	2	1	/	3	2	2	/	/	19	53
10.1002/jmri.27532	Yan et al. 2021 [39]	2	1	/	/	3	1	/	1	2	2	/	3	2	2	/	/	19	53
10.5603/RPOR.a2021.0092	Gonzàlez-Viguera et al. 2021 [23]	1	/	/	/	3	/	1	/	1	/	/	-5	/	2	/	/	3	8
10.3389/fonc.2021.710649	Chen et al. 2021 [29]	1	1	/	/	3	1	/	1	2	2	/	-5	2	2	/	/	10	28
10.1016/j.radonc.2021.08.023	Peeken et al. 2021 [50]	2	1	/	/	3	1	1	/	2	2	/	3	2	2	/	2	21	58
10.1186/s40644-021-00438-y	Zhao et al. 2022 [31]	2	/	/	/	3	/	/	/	2	/	/	2	/	2	/	4	15	42
10.1007/s40042-021-00360-3	Sheen et al. 2022 [17]	2	1	/	/	3	/	1	/	2	2	/	-5	/	2	/	1	9	25
10.1007/s13304-021-01074-8	Yang et al. 2022 [22]	2	/	/	/	3	1	/	1	1	2	/	2	/	2	/	/	14	39
10.1007/s00330-021-08221-0	Liu et al. 2022 [33]	2	/	/	/	3	1	/	1	2	2	/	4	2	2	/	/	19	53
10.3390/tomography8010028	Tomaszewski et al. 2022 [53]	/	/	/	/	3	1	1	1	2	/	/	2	2	2	/	/	14	39
10.3389/fonc.2022.897676	Liang et al. 2022 [13]	2	1	/	/	3	/	1	1	1	2	/	4	2	2	/	4	23	64
10.2478/raon-2022-0013	Giraudo et al. 2022 [45]	2	1	/	/	3	1	1	/	1	/	/	-5	/	2	/	/	6	17
10.1002/mp.15603	Escobar et al. 2022 [15]	2	/	/	/	3	/	1	/	2	/	/	-5	/	2	/	4	9	25
10.3389/fonc.2022.899180	Thrussell et al. 2022 [58]	2	/	/	1	3	/	1	/	1	/	/	-5	/	2	/	4	9	25
10.1002/jmri.28021	Fadli et al. 2022 [28]	1	1	/	/	3	1	1	1	1	/	/	-5	2	2	/	2	10	28
10.1186/s12880-022-00859-6	Miao et al. 2022 [49]	2	/	/	/	3	/	1	/	1	/	/	2	/	2	/	4	15	42
10.1186/s12880-022-00876-5	Liu et al. 2022 [41]	/	/	/	/	3	/	/	/	2	/	/	-5	/	2	/	4	6	17
10.3389/fonc.2022.879553	Giraudo et al. 2022 [20]	1	1	/	/	3	/	1	/	1	/	/	-5	/	2	/	2	6	17
10.1002/jmri.28518	Yang et al. 2022 [38]	1	1	/	/	3	1	1	1	2	1	/	3	/	2	/	/	16	44
10.1002/mp.16136	Hu et al. 2022 [14]	1	1	/	/	3	1	1	1	2	2	/	3	/	2	/	/	17	47
10.1007/s11307-022-01751-z	Yue et al. 2022 [59]	2	1	/	/	3	1	1	1	1	/	/	2	/	2	/	/	14	39
10.1002/jmri.28160	Yang 2022 [25]	1	1	/	/	3	1	/	1	2	2	/	2	2	2	/	2	19	53
10.3390/jcm12010279	Annovazzi et al. 2022 [27]	2	/	/	/	3	1	1	1	1	/	/	-5	/	2	/	4	10	28
10.1007/s11307-023-01803-y	Fields et al. 2023 [51]	1	/	/	/	3	/	/	/	2	/	/	-5	/	2	/	/	3	8
10.1007/s00330-022-09104-8	Crombé et al. 2023 [46]	1	/	/	/	/	1	1	/	2	/	/	-5	/	2	/	/	2	6
10.1002/jmri.28331	Lee et al. 2023 [30]	1	1	/	/	3	1	1	/	1	/	/	-5	2	2	/	/	7	19
10.1088/2057-1976/acc33a	Aouadi et al. 2023 [60]	2	/	/	/	3	/	1	/	2	/	/	4	/	2	/	2	16	44
10.3390/cancers15072150	Foreman et al. 2023 [48]	2	1	/	/	3	1	/	/	2	/	/	3	/	2	/	2	16	44
10.1093/jamiaopen/ooad025	Casale et al. 2023 [34]	2	/	/	/	3	/	1	/	/	/	/	2	/	2	/	2	12	33
10.1177/02841851231179933	Zhu et al. 2023 [43]	1	/	/	/	3	/	1	/	1	/	/	2	/	2	/	2	12	33

## Discussion

Imaging has a prominent role in diagnosis and in treatment decision making in patients with STS. When an STS is clinically suspected, the role of diagnostic imaging and multidisciplinary discussions are essential. Ultrasound is often the first-approach modality, but MRI is mandatory to characterize the lesion, assess its anatomical limits and therefore guide treatment decision [3]. Also, some STS features seen on MRI may predict the grade of malignancy of the lesions [62], recurrences [63, 64], post-treatment oedema and seroma [65]. Thoracic CT or PET-CT are used to assess secondary lesions. Imaging is also important to guide a biopsy, which may be necessary to confirm the exact histological nature of the lesion. Recently, the development of new imaging techniques, such as whole-body MRI, hybrid PET/MRI, diffusion weighted MRI, dynamic contrast MRI and advances in artificial intelligence have greatly enhanced the radiologist role in tumour grading and staging assessment.

However, the only use of imaging has still a very limited accuracy and therefore a histological confirmation is often needed. In order to improve imaging accuracy, several new imaging techniques were proposed in latest years. One of these techniques, radiomics, has shown promising results. It may be associated with the notion of radiogenomics, positing that imaging characteristics correlate with genetic signatures [6]. This method surpasses conventional imaging by being non-invasive, objective, and cost-effective. Importantly, radiomics could provide complementary information to histopathology and molecular biomarkers, enhancing tumour evaluation and aiding in personalised treatment strategies.

In recent advancements within oncologic imaging, radiomics has extended its utility to the field of STSs. Our review elucidates the versatile applications of radiomic analysis in STSs, showcasing interesting results across various critical domains. Notably, radiomics has demonstrated significant potential in aiding with diagnosis, facilitating risk stratification, predicting grading or genomic status, and evaluating treatment responses in STSs.

Our review underscores the potential of radiomics to complement and augment conventional imaging approaches. We have observed that radiomics, with its high-throughput data analysis capabilities, can provide deeper insights into the imaging characteristics of STSs. This integration of radiomic techniques with traditional imaging approaches marks a significant advancement in the field.

Albeit radiomics discoveries have shown promises, more work is needed to ensure these results can be replicated with a satisfactory degree of proof by enrolling larger patient datasets. The heterogeneity of features, the variability of acquisition protocols and multiple image modalities further complicate the generalizability of radiomics observations. Moreover, independent validation of proposed predictive models often is lacking, making extensive radiomics studies in STSs challenging. Additionally, the complexities inherent in radiomics may challenge its integration into routine clinical practice, suggesting a need for tailored training and resources to facilitate its use among clinicians and radiologists. An improved implementation of the latest guidelines [12, 56, 66–68] and increased knowledge of radiomics will be necessary to enhance the quality of sarcoma radiomics studies and to enable their implementation in the clinical setting.

## Conclusion

Our review highlights radiomics as a potential adjunct to conventional STS methodologies, enhancing diagnostic and treatment strategies. Yet, the limitations of the current approach necessitate further studies. Additionally, the complexities inherent in radiomics may challenge its integration into routine clinical practice. Future investigations should focus on validating radiomics' clinical application and establishing its role in STS management practices.

## Data Availability

All data are available on PubMed/MEDLINE and Scopus.

## Abbreviations

STS: Soft tissue sarcoma
CT: Computed tomography
PET: Positron Emission Tomography
MRI: Magnetic Resonance Imaging
PRISMA: Preferred Reporting Items for Systematic Reviews and Meta-Analyses
RQS: Radiomics Quality Score
DWI: Diffusion Weighted Imaging
ADC: Apparent Diffusion Coefficient
T2FS: Fat-Suppressed T2-weighted
ROC: Receiver Operating Characteristic
AUC: Area Cnder the receiver operator characteristic curve
LASSO: Least Absolute Shrinkage and Selection Operator
SVM: Support Vector Machine
RF: Random Forest
